# Development of a Panel of Biomarkers for Differential Diagnosis of Multiple Sclerosis

**DOI:** 10.1134/S160767292460060X

**Published:** 2024-11-11

**Authors:** L. A. Ovchinnikova, S. S. Dzhelad, T. O. Simaniv, M. N. Zakharova, Y. A. Lomakin, A. G. Gabibov, S. N. Illarioshkin

**Affiliations:** 1 Shemyakin–Ovchinnikov Institute of Bioorganic Chemistry, Russian Academy of Sciences, Moscow, Russia; 2 Research Center of Neurology, Moscow, Russia; 3 Moscow State University, Moscow, Russia

**Keywords:** multiple sclerosis, MS, autoantibodies, EBV, Epstein–Barr virus, demyelinating diseases

## Abstract

Demyelinating diseases are a group of heterogeneous pathologies that affect the nervous system and reduce the quality of life. One of such diseases is multiple sclerosis (MS), an inflammatory autoimmune neurodegenerative disease of the central nervous system (CNS). At the initial stages, MS can mimic some infectious, neoplastic, genetic, metabolic, vascular, and other pathologies. Accurate differential diagnosis of this disease is important to improve the quality of life of patients and reduce possible irreversible damage to the central nervous system. In this work, we confirmed the possibility of using our previously proposed candidate panel of MS biomarkers to distinguish MS from neuromyelitis optica spectrum disorder (NMOSD) and amyotrophic lateral sclerosis (ALS). We have shown that our proposed panel (SPTAN1_601-644_ + PRX_451-494_ + PTK6_301-344_ + LMP1_285-330_) allows us to distinguish MS from ALS (AUC = 0.796) and NMOSD (AUC = 0.779).

## INTRODUCTION

Demyelinating diseases of the central nervous system (CNS) are a heterogeneous group of pathologies, the common feature of which is damage to the nervous tissue with the involvement of oligodendrocytes, leading to various neurological disorders. Often, symptoms specific to each individual disease may not appear immediately, thereby complicating differential diagnosis and delaying the start of therapy. One of these diseases is multiple sclerosis (MS), an immunity-mediated disease of the central nervous system (CNS), in which the myelin sheath of the nerve fiber is damaged [1]. Over time, this disease inevitably progresses, which reflects a partial transition from a predominantly localized acute injury to widespread inflammation and neurodegeneration, coupled with a failure of compensatory mechanisms such as neuroplasticity and remyelination [2]. However, in the initial stages, the symptoms of MS can be confused with the manifestation of some infectious, neoplastic, genetic, metabolic, vascular, and other idiopathic inflammatory demyelinating disorders [3]. MS can be diagnosed according to McDonald’s criteria based on MRI after the first episode of clinical attack [4], when the inflammatory lesion of the myelin sheath is irreversible. Diseases that require differential diagnosis with MS include neuromyelitis optica spectrum disorders (NMOSD), previously classified as MS variants but requiring fundamentally different therapy [5, 6]. In case of NMOSD, the foci of inflammation are localized mainly along the optic nerve and spinal cord. This disease is also characterized by the presence of antibodies to aquaporin-4. Primary neurodegenerative diseases include amyotrophic lateral sclerosis (ALS). This pathology is characterized by specific symptoms associated with damage to motor neurons located both in the motor cortex and in the nuclei of the cranial nerves and the anterior horns of the spinal cord. However, diagnosing ALS is often difficult, especially in the early stages of the disease [7].

Previously, based on the level of serum antibodies to potential autoimmune aggression targets, we proposed the following candidate peptide biomarkers of MS: protein tyrosine kinase 6 (PTK6_301–344_), periaxin (PRX_451–494_), and alpha-II-spectrin (SPTAN1_601–644_). When an additional antigen, the latent membrane protein 1 (LMP1) of the Epstein–Barr virus (EBV), the most likely trigger of MS, is added to this panel, the diagnostic sensitivity of the latter increases. The aim of this study was to determine the possibility of using the proposed panel of antigens in samples of patients with neurological diseases other than MS. Such validation will make it possible to determine the predictive value of autoantigen markers on the basis of the titer of antigen-specific autoreactive antibodies in patients with MS, but not in patients with other diseases that are also characterized by CNS damage.

## MATERIALS AND METHODS

The amount of antigen-specific antibodies to the studied antigens in blood serum of patients with MS (*n* = 28), ALS (*n* = 14), and NMOSD (*n* = 5) was determined by the enzyme-linked immunosorbent assay (ELISA). Three measurements were taken for each patient.

The diagnostic accuracy of optimal combinations of serum autoreactive IgG levels was determined using the CombiROC analysis [8]. The best biomarker combinations were identified by analyzing the receiver operating characteristic (ROC) curves and calculating the sensitivity (SE) and specificity (SP) of all possible marker combinations (http://CombiROC.eu). The significance of the obtained values was evaluated using the permutation test.

## RESULTS AND DISCUSSION

**Fig. 1.**
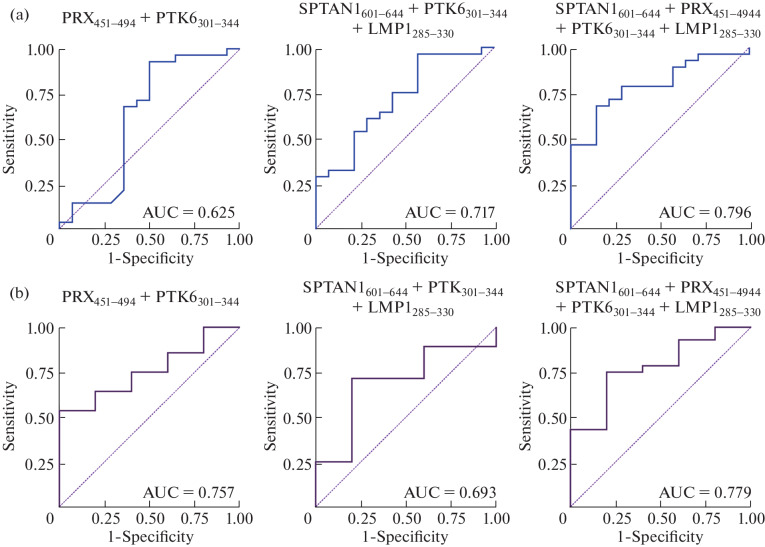
ROC analysis of selected combinations of autoantigens and viral peptide of Epstein–Barr virus for differential diagnosis of MS from other neurodegenerative diseases. (a) ROC curves obtained by comparing the effectiveness of candidate panels for differential diagnosis of MS and amyotrophic lateral sclerosis (ALS). (b) ROC curves obtained by comparing the effectiveness of candidate panels for differential diagnosis of multiple sclerosis and neuromyelitis optica spectrum disorders (NMOSD). AUC—area under curve.

To create an effective panel of candidate biomarkers, we selected the following combinations of peptide targets: (1) PRX_451-494_ + PTK6_301-344_, (2) SPTAN1_601-644_ + PTK6_301-344_ + LMP1_285-330_, and (3) SPTAN1_601-644_ + PRX_451-494_ + PTK6_301-344_ + LMP1_285-330_. These autoantigen and viral peptides were used to determine the level of specific antibodies in the total IgG pool of the peripheral blood of patients with MS, NMOSD, and ALS. On the basis of the results of this analysis, ROC curves, which characterize the accuracy of the used panel of autoantigens for differential diagnosis of the listed neurodegenerative diseases, were constructed (Fig. 1). The characteristics obtained for these ROC curves are presented in Table 1. It is evident from the data obtained that the use of two (SPTAN1_601-644_ + PTK6_301-344_) or three (SPTAN1_601-644_ + PRX_451-494_ + PTK6_301-344_) autoantigens in combination with the viral protein (LMP1_285-330_) allow differentiating MS from ALS (AUC > 0.7, Fig. 1a). In turn, to differentiate MS from NMOSD, a panel of two autoantigens without the viral peptide (PRX_451-494_ + PTK6_301-344_) or containing all four antigens (SPTAN1_601-644_ + PRX_451-494_ + PTK6_301-344_ + LMP1_285-330_) can be used (Fig. 1b). Moreover, the greatest value of the area under curve (AUC), characterizing higher recognition accuracy, was obtained for the panel containing all four biomarkers (SPTAN1_601-644_ + PRX_451-494_ + PTK6_301-344_ + LMP1_285-330_) when discriminating both between MS and ALS (AUC = 0.796) and between MS and NMOSD (AUC = 0.779).

**Table 1.** Indices obtained from the results of ROC analysis for different combinations of autoantigens and viral peptide LMP1

Comparison groups	Combination of biomarkers	AUC	ACC	SE	SP
MS and ALS	PRX_451-494_ PTK6_301-344_	0.625	0.786	0.929	0.500
	SPTAN1_601-644_ PTK6_301-344_ LMP1_285-330_	0.717	0.786	0.964	0.429
	SPTAN1_601-644_ PRX_451-494_ PTK6_301-344_ LMP1_285-330_	0.796	0.738	0.679	0.857
MS and NMOSD	PRX_451-494_ PTK6_301-344_	0.757	0.606	0.536	1
	SPTAN1_601-644_ PTK6_301-344_ LMP1_285-330_	0.693	0.727	0.714	0.800
	SPTAN1_601-644_ PRX_451-494_ PTK6_301-344_ LMP1_285-330_	0.779	0.758	0.750	0.800

MS—multiple sclerosis, ALS—amyotrophic lateral sclerosis, NMOSD—neuromyelitis optica spectrum disorder, AUC—area under curve, ACC—diagnostic accuracy, SE—sensitivity, SP—specificity.

**Fig. 2.**
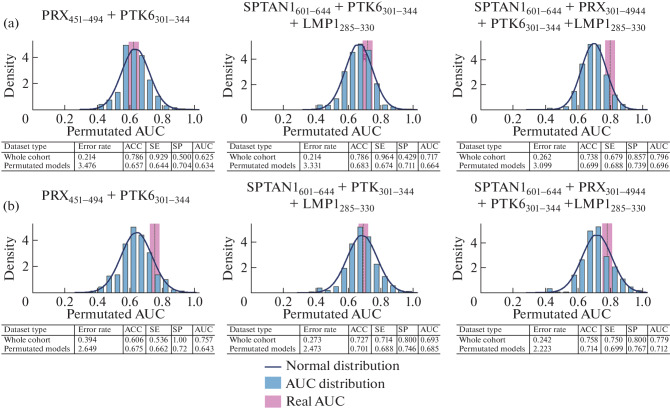
Permutation test (rearrangement test). (a) Distinguishing MS from ALS (amyotrophic lateral sclerosis). (b) Distinguishing multiple sclerosis from neuromyelitis optica spectrum disorders (NMOSD). AUC—area under curve, ACC—diagnostic accuracy, SE—sensitivity, SP—specificity.

Next, to more reliably assessment of the significance of the obtained results and confirm the effectiveness of the proposed panels of potential MS markers, we performed a statistical test with permutation of the obtained values of antigen-specific antibody titers for each donor (Fig. 2). This statistical test involves multiple random redistribution of the set of observed data for a more accurate assessment of the significance of the obtained result. According to the results of this test, the obtained AUC values for the most effective panel (SPTAN1_601-644_ + PRX_451-494_ + PTK6_301-344_ + LMP1_285-330_) drastically differ from the values for the redistributed samples, which indicates that the proposed candidate panel of four MS biomarkers can be further used. It is clear that, in the case of using only two (PRX_451-494_ + PTK6_301-344_) or three (SPTAN1_601-644_ + PTK6_301-344_ + LMP1_285-330_) markers, the obtained AUC values are nonsignificant, since they do not differ significantly from the AUC values for a random distribution of the data set.

## CONCLUSIONS

MS is an extremely heterogeneous disease, which can currently be reliably diagnosed only after the first episode of a clinical attack. However, even after such an episode, the symptomatic manifestations of MS are often similar to symptoms of other neurological diseases. The proposed candidate panel of biomarkers (SPTAN1_601-644_ + PRX_451-494_ + PTK6_301-344_ + LMP1_285-330_) makes it possible to differentiate MS from other diseases studied (AUC = 0.796 for ALS and AUC = 0.779 for NMOSD), which indicates its high potential for diagnosing MS. However, the diagnostic value of the detected antigens requires further studies, and we hope that the identified peptides may become a convenient, cost-effective, and significant serological biomarker for diagnosing multiple sclerosis. We expect that further long-term studies, including cerebrospinal fluid analysis, will confirm our conclusions and help to establish the role of the identified antigens in the pathogenesis of multiple sclerosis.
